# Supplementary material for: The Salmonella virulence protein MgtC promotes phosphate uptake inside macrophages
Source: Nat Commun. 2019 Jul 25;10:3326. doi: 10.1038/s41467-019-11318-2 (PMC6658541; doi:10.1038/s41467-019-11318-2)
Supplement: Supplementary file 1 — Supplementary Information [file 41467_2019_11318_MOESM1_ESM.pdf]

Supplementary Information

A *Salmonella* virulence protein promotes phosphate uptake inside macrophages

Choi et al.

Supplementary Note

Identification of a region or residue of MgtC required for PhoR interaction

MgtC has the membrane-embedded N-terminal domain with five transmembrane helices and cytoplasmic C-terminal domain. We found that the C-terminal cytoplasmic domain of MgtC is not sufficient for PhoR interaction because a T18-fused *mgtC* fragment corresponding to the C-terminal cytoplasmic domain (positions 130-231) lost the ability to interact with coexpressed T25-PhoR (Supplementary Fig. 2).

Previously, the Asn92 residue at the fourth transmembrane helix of the MgtC protein was reported to be required for interacting with F_1F_o ATP synthase[1] (Supplementary Fig. 2a). Because the data presented above suggests that the N-terminal transmembrane region of MgtC is required for PhoR interaction, we then wondered whether the Asn92 residue is involved in PhoR interaction. To explore this idea, we constructed a C-terminally T18-fused *mgtC* variant with an Asn92 to Thr substitution and coexpressed it with T25-PhoR. The MgtC-T18 variant with the Asn92 to Thr substitution failed to interact with T25-PhoR (Supplementary Fig. 2c) and a chromosomal *mgtC* variant with the same substitution did not increase mRNA levels of the *phoE* gene (Supplementary Fig. 2d), indicating that the Asn92 residue of the MgtC protein is required for PhoR interaction, in addition to the previously identified interaction with F_1F_o ATP synthase[1].

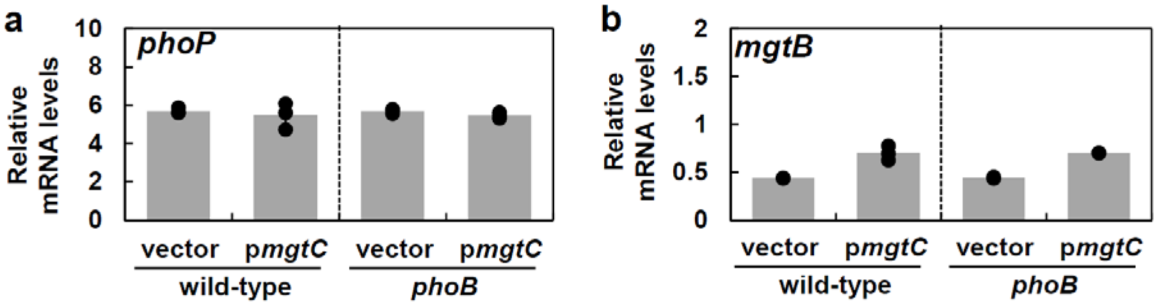

Supplementary Fig. 1 *mgtC* overexpression has no effect on mRNA levels of the *phoP* and *mgtB* genes, related to Fig. 1.
(**a-b**) Relative mRNA levels of the *phoP* (**a**) and *mgtB* (**b**) genes in *Salmonella* strains listed in Fig. 1. Bacteria were grown for 3 h in N-minimal media containing 10 mM Mg^{2+} and then for an additional 1 h in the same media containing 0.5 mM Mg^{2+} and 0.25 mM IPTG. Data are represented as mean ± SD (n=3). Relative mRNA levels represent (target RNA/*rrsH* RNA) × 10000.

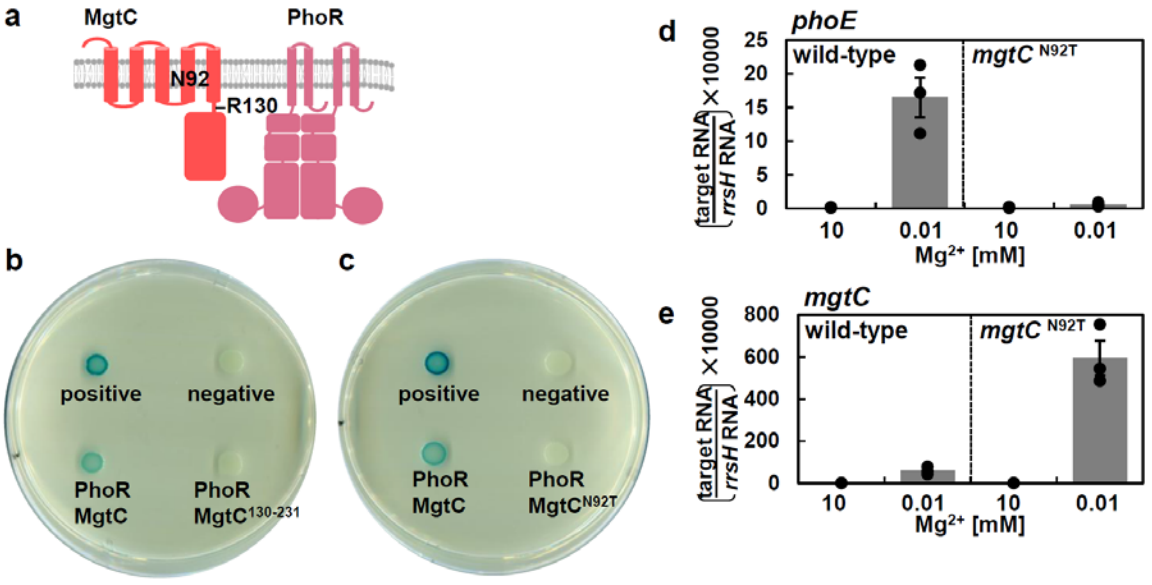

Supplementary Fig. 2 The N-terminal transmembrane domain and Asn92 residue in the fourth transmembrane helix of the MgtC protein are required for PhoR interaction, related to Fig. 2.

(**a**) Schematic representation of the MgtC protein including Asn92 residue in the fourth transmembrane helix and Arg130 residue in the boundary between the N- and C-terminal regions.

(**b**) Bacterial two-hybrid assay between the $\text{MgtC}^{\text{wild-type}}$ or $\text{MgtC}^{130\text{-}231}$ and PhoR proteins.

(**c**) Bacterial two-hybrid assay between the $\text{MgtC}^{\text{wild-type}}$ or $\text{MgtC}^{\text{N92T}}$ and PhoR protein. *Escherichia coli* strain BTH101 harboring two plasmids (pUT18 and pKT25 derivatives) expressing the C-terminal fusions of the *cyaA* T18 fragment to the coding regions of the wild-type *mgtC*, $mgtC^{130\text{-}231}$, or $mgtC^{\text{N92T}}$ gene and N-terminal fusion of the *cyaA* T25 fragment to the *phoR* coding region, or the pKT25 empty vector (negative) as indicated. Cells expressing both pUT18-*mgtC* and pKT25-*mgtR* are spotted as a positive control [2]. Cells were spotted onto LB plates containing 80 μM X-gal and 0.1 mM IPTG and incubated at 30°C for 40 h. Blue colored colonies indicate a positive interaction.

(**d-e**) Relative mRNA levels of the *phoE* (**d**) and *mgtC* (**e**) genes in *Salmonella* strains with the wild-type *mgtC* or the Asn92 to Thr-substituted *mgtC* ($mgtC^{\text{N92T}}$, EN551). Bacteria were grown for 5 h in N-minimal media containing 10 mM or 0.01 mM Mg^{2+}. Data are represented as mean ± SEM (n=3). Relative mRNA levels represent (target RNA/*rrsH* RNA) × 10000.

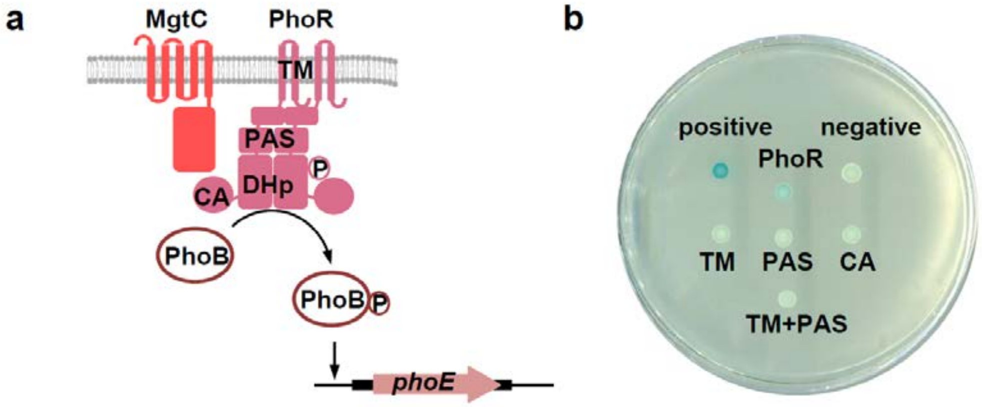

Supplementary Fig. 3 None of the domains of the PhoR protein are sufficient for MgtC interaction, related to Fig. 3.
(**a**) Schematic representation of the domain structure of the PhoR protein including a TM (TransMembrane) domain, a PAS (Per-Arnt-Sim) domain, a DHp (Dimerization Histidine phosphotransfer) domain, and a CA domain (Catalytic/ ATP-binding).
(**b**) Bacterial two-hybrid assay between the MgtC and full-length PhoR protein or its domains. *Escherichia coli* BTH101 strains harboring two plasmids (pUT18 and pKT25 derivatives) expressing the C-terminal fusion of the *cyaA* T18 fragment to the *mgtC* coding region and N-terminal fusions of the *cyaA* T25 fragment to either the coding regions of the full-length *phoR* (PhoR), *phoR*$_{1\text{-}60}$ (TM), *phoR*$_{61\text{-}180}$ (PAS), *phoR*$_{181\text{-}431}$ (CA), *phoR*$_{1\text{-}180}$ (TM+PAS), and *mgtR* (positive) genes or the pKT25 empty vector (negative) were indicated. Cells were spotted onto LB plates containing 80 µM X-Gal and 0.1 mM IPTG and incubated at 30°C for 40 h. Blue colored colonies indicate a positive interaction.

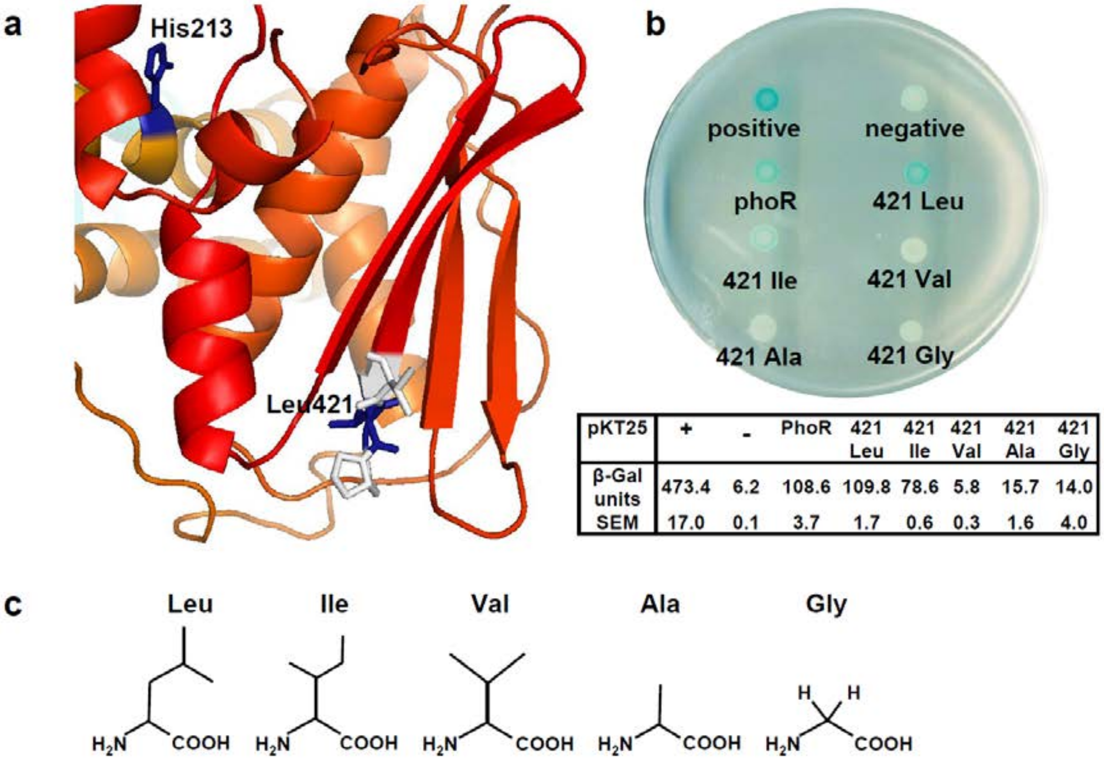

pKT25	+	-	PhoR	421 Leu	421 Ile	421 Val	421 Ala	421 Gly
β-Gal units	473.4	6.2	108.6	109.8	78.6	5.8	15.7	14.0
SEM	17.0	0.1	3.7	1.7	0.6	0.3	1.6	4.0

Supplementary Fig. 4 The large and hydrophobic side chain at position 421 in PhoR is required for MgtC interaction, related to Fig. 3.
(**a**) The detailed top view of a PhoR region including Leu421 in the CA domain and His213 in the DHp domain.
(**b**) Bacterial two-hybrid assay between the MgtC and full-length PhoR protein or its derivatives. *Escherichia coli* strain BTH101 harboring two plasmids (pUT18 and pKT25 derivatives) expressing the C-terminal fusion of the *cyaA* T18 fragment to the *mgtC* coding region and N-terminal fusions of the *cyaA* T25 fragment to either the coding regions of the wild-type *phoR* (PhoR), or its variants with the *phoR*$_{1\text{-}421}$ (421 Leu), *phoR*$_{1\text{-}421}$ variants where leucine 421 was substituted by isoleucine (421 Ile), valine (421 Val), alanine (421 Ala), or glycine (421 Gly), and *mgtR* (positive) genes or the pKT25 empty vector (negative) as indicated. Cells were spotted onto LB plates containing 80 μM X-Gal and 0.1 mM IPTG and incubated at 30°C for 40 h. Blue colored colonies indicate a positive interaction. The average β-galactosidase activities (β-Gal units) are shown below with the SEM (n=3).
(**c**) Comparison of the side chains in leucine, isoleucine, valine, alanine, and glycine.

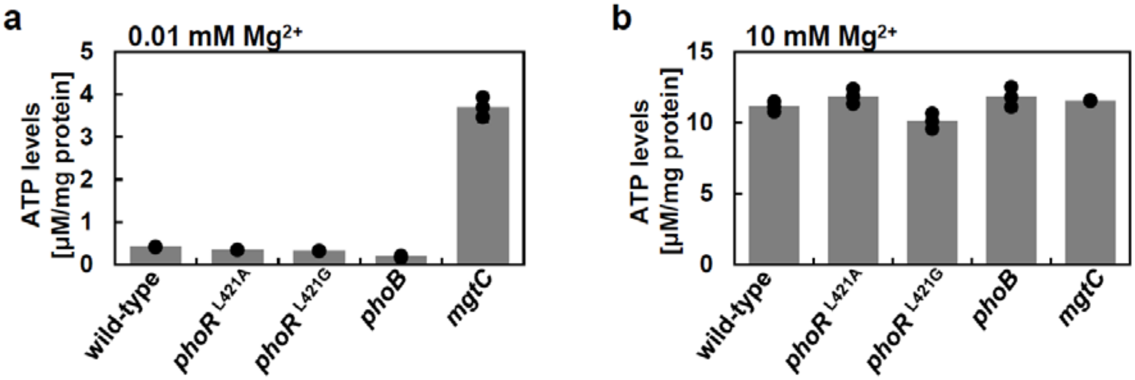

Supplementary Fig. 5 Leucine 421 to alanine or glycine substitution in PhoR does not affect intracellular ATP levels, related to Fig. 4.
(**a-b**) Intracellular ATP levels of wild-type *Salmonella* (14028s), the *phoR* chromosomal mutant with Leu421 replaced by Ala codon (EN949) or Gly codon (EN991), the *phoB* deletion mutant (KK10), and the *mgtC* deletion mutant (EL4) grown for 5 h in N-minimal media containing 0.01 mM (**a**) or 10 mM Mg^{2+} (**b**). Intracellular ATP levels correspond to micromole of ATP per mg of total protein (mean ± SD, n=3).

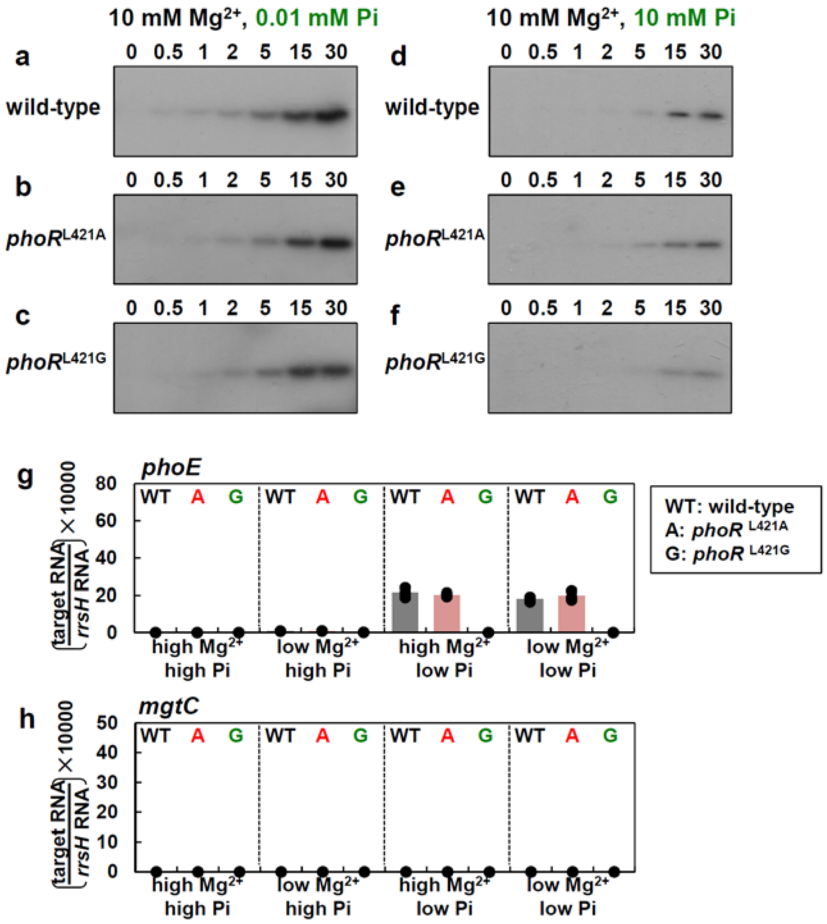

Supplementary Fig. 6 Leucine 421 to alanine substitution in *phoR* has no effect on low Pi-mediated PhoR autophosphorylation and is functional to mediate the low phosphate-induced mRNA increase of the *phoE* gene, related to Fig. 4.

(**a-f**) Autophosphorylation assay. Levels of PhoR-P following incubation of membrane vesicles prepared from *Salmonella* strains with the wild-type *phoR* (14028s, **a** and **d**), the Leu421 to Ala-substituted *phoR* (EN949, **b** and **e**), or the Leu421 to Gly-substituted *phoR* gene (EN991, **c** and **f**) grown in N-minimal media containing 0.01 mM (**a-c**) or 10 mM (**d-f**) Pi with [γ^{32}P] ATP at the indicated times. Bacteria were grown for 5 h in N-minimal media containing 10 mM Mg^{2+} and either 0.01 mM or 10 mM Pi and membrane vesicles were prepared as described in Methods. Supplementary Figs. 4d, 4e, and 4f are identical to Figs. 4e, 4f, and 4g, respectively.

(**g-h**) Relative mRNA levels of the *phoE* (**g**) and *mgtC* (**h**) genes in *Salmonella* strains with the wild-type *phoR* (WT (EL4), black bar), the Leu421 to Ala-substituted *phoR* (A (SM085), red bar), or the Leu421 to Gly-substituted *phoR* gene (G (SM086), green bar) in the *mgtC* deletion background. Bacteria were grown for 5 h in N-minimal media containing combinations of 10 mM (high Mg^{2+}) or 0.01 mM (low Mg^{2+}) Mg^{2+} and 10 mM (high Pi) or 0.01 mM (low Pi) Pi. Data are represented as mean ± SD (n=3). Relative mRNA levels represent (target RNA/*rrsH* RNA) × 10000.

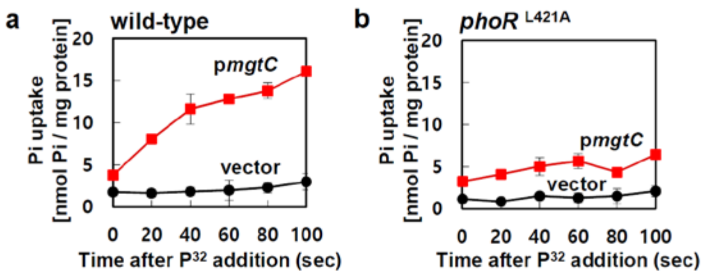

Supplementary Fig. 7 Heterologous expression of the *mgtC* gene promotes phosphate uptake, related to Fig. 4.
(**a**-**b**) phosphate transport assay using whole cells of either wild-type (**a**) or *phoR* ^{L421A} (**b**) *Salmonella* harboring a plasmid with the *mgtC* gene (red) or the empty vector (black). Bacteria were grown for 3 h in N-minimal media containing 10 mM Mg^{2+} and then for an additional 1 h in the same media containing 0.5 mM Mg^{2+} and 0.25 mM IPTG. Levels of radioactive orthophosphate accumulated in cells were determined over time by liquid scintillation counting as described in Methods (mean ± SD, n=3).

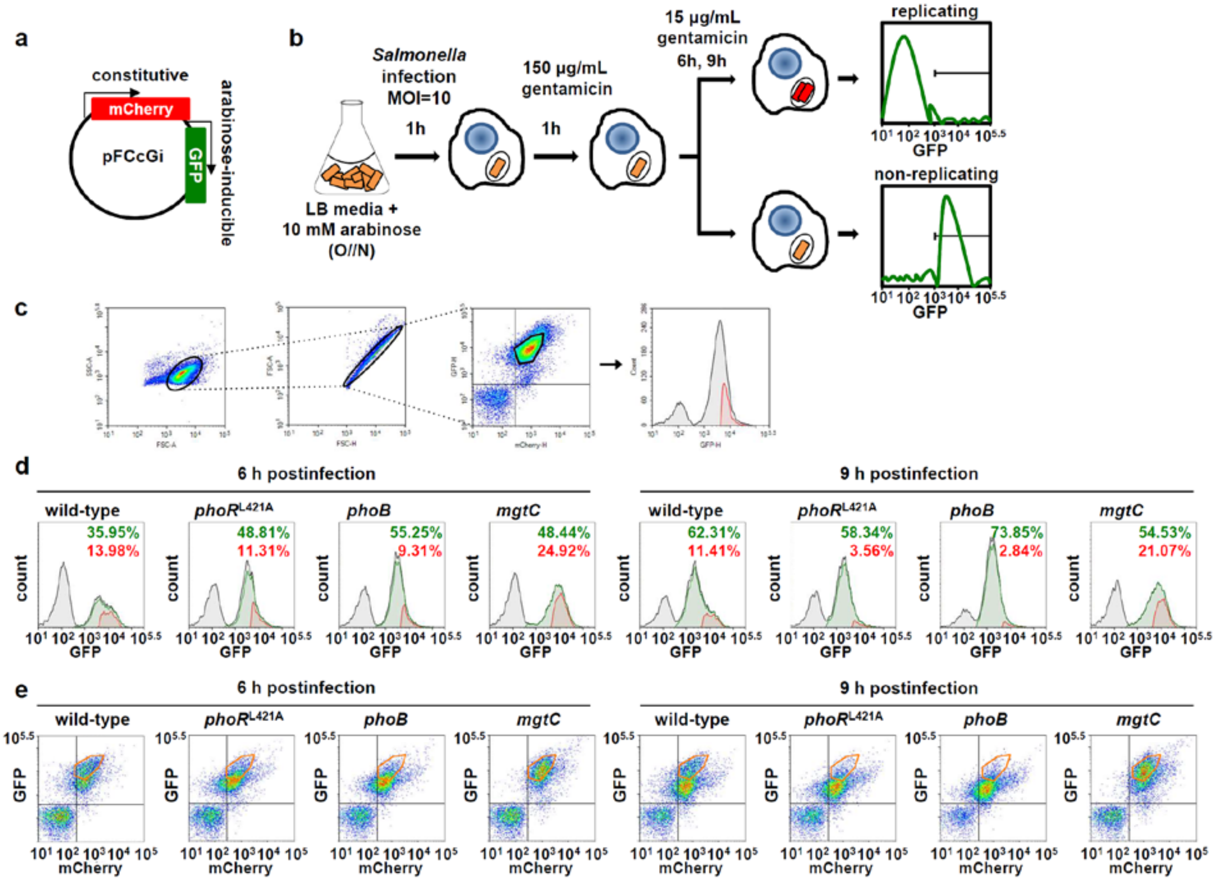

f

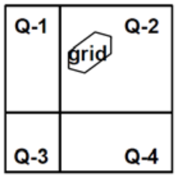

	6h postinfection				9h postinfection			
Gate	wild-type	*phoR* L421A	*phoB*	*mgtC*	wild-type	*phoR* L421A	*phoB*	*mgtC*
Q-1	1455	2710	2065	263	2193	2283	2225	243
Q-2	10335	14643	16575	14533	18693	17503	22155	16360
Q-3	17493	12323	11165	14775	8733	9750	5163	12920
Q-4	268	325	195	430	383	465	458	478
grid	4163	3393	2793	7475	3423	1068	853	6320

Supplementary Fig. 8 Leucine 421 to alanine substitution in *phoR* decreases the formation of non-replicating *Salmonella* inside macrophages, related to Fig. 5.
(**a**) Schematic representation of the pFCcGi plasmid.
(**b**) Schematic representation of measuring non-replicating *Salmonella* inside macrophages by flow cytometry.
(**c**) Gating strategy for flow cytometry analysis. Cells were firstly gated with FSC/SSC dot blot to select live bacteria, followed by second gating (FSC-H versus FSC-A) to select single cells. Then, mCherry/GFP plots were analyzed to select bacteria that emit high levels of red fluorescence and green fluorescence simultaneously as shown in a hexagon grid.
(**d**) Histograms of *Salmonella* strains expressing GFP inside J774 A.1 macrophages. Histograms indicate the GFP fluorescence intensities of strains listed in Figure 5 at 6 or 9 h postinfection, with gates showing the fraction of the population exhibiting high and

low levels of GFP. Non-replicating GFP^{High} cells are indicated as red histograms and cells in the Q2 area are indicated as green histograms. Bacterial fractions in the grid and Q2 area are indicated in red and green respectively. The percentage of cells expressing high levels of GFP inside macrophages at the indicated times was calculated based on the following formula: (*Salmonella* inside the grid)/(total number of *Salmonella* in Q-2 area).

(**e**) Detection of mCherry and GFP fluorescence in *Salmonella* population harboring pFCcGi plasmid (n = 30,000 cells) inside J774 A.1 macrophages at 6 and 9 h postinfection in the absence of arabinose. Orange grids were drawn based on GFP^{High} populations of wild-type *Salmonella* at 1 h postinfection and used as a grid to chase the percentage of remaining non-replicating cells at indicated times. Data are representative of three independent experiments.

(**f**) Absolute numbers of events in each fraction presented in (**e**).

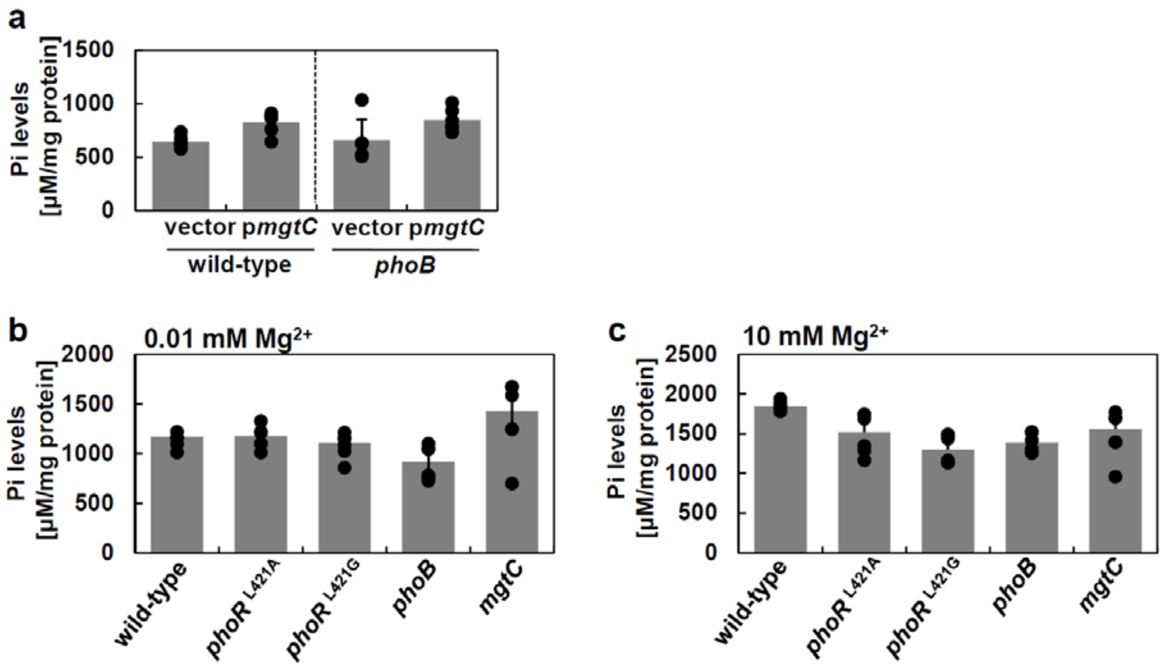

Supplementary Fig. 9 Heterologous *mgtC* expression or replacing leucine 421 by alanine or glycine in *phoR* does not alter steady-state levels of intracellular phosphate in cells grown in MOPS media containing 0.5 mM Pi, related to Figs. 1, 4, and 5.
(**a**) Intracellular phosphate levels of strains listed in Figs. 1c-1h. Bacteria were grown for 3 h in MOPS media containing 0.5 mM Pi and 10 mM Mg^{2+} and then for an additional 1 h in the same media containing 0.5 mM Pi, 0.5 mM Mg^{2+}, and 0.25 mM IPTG. Intracellular phosphate levels correspond to micromole of phosphate per mg of total protein.
(**b**-**c**) Intracellular phosphate levels of wild-type *Salmonella* (14028s), the *phoR* chromosomal mutant with Leu421 replaced by Ala codon (EN949) or Gly codon (EN991), the *phoB* deletion mutant (KK10), and the *mgtC* deletion mutant (EL4) grown for 5 h in MOPS media containing 0.5 mM Pi and either 0.01 mM (**b**) or 10 mM Mg^{2+} (**c**). Intracellular phosphate levels correspond to micromole of phosphate per mg of total protein (mean ± SD, n=6).

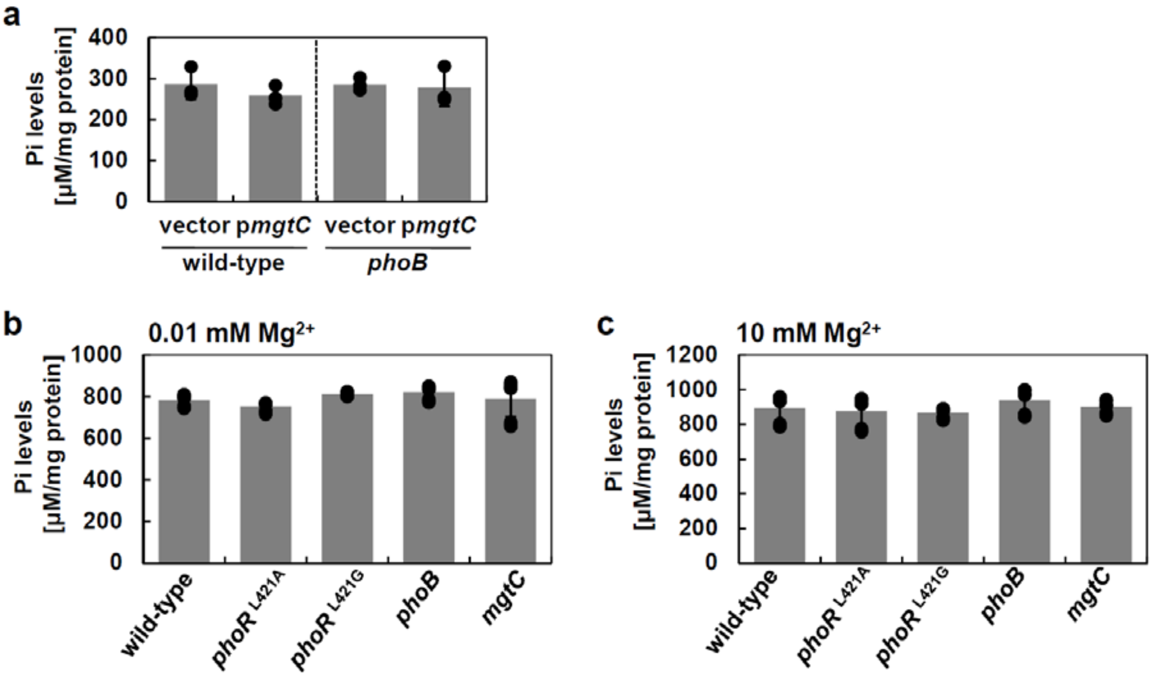

Supplementary Fig. 10 Heterologous *mgtC* expression or replacing leucine 421 by alanine or glycine in *phoR* does not alter steady-state levels of intracellular phosphate in cells grown in N-minimal media containing 10 mM KH_2PO_4, related to Figs. 1, 4, and 5. (**a**) Intracellular phosphate levels of strains listed in Figs. 1c-1h. Bacteria were grown for 3 h in N-minimal media containing 10 mM Mg^{2+} and then for an additional 1 h in the same media containing 0.5 mM Mg^{2+} and 0.25 mM IPTG. Intracellular phosphate levels correspond to micromole of phosphate per mg of total protein (mean ± SD, n=3). (**b**-**c**) Intracellular phosphate levels of wild-type *Salmonella* (14028s), the *phoR* chromosomal mutant with Leu421 replaced by Ala codon (EN949) or Gly codon (EN991), the *phoB* deletion mutant (KK10), and the *mgtC* deletion mutant (EL4) grown for 5 h in N-minimal media containing 0.01 mM (**b**) or 10 mM Mg^{2+} (**c**). Intracellular phosphate levels correspond to micromole of phosphate per mg of total protein (mean ± SD, n=9).

Supplementary Table 1. Bacterial strains and plasmids used in this study

Strain or plasmid	Description	Reference or source
S. enterica **serovar Typhimurium**		
14028s	wild-type	[3]
MS7953s	*PhoP7953*::Tn10	[4]
KK131	14028s/pBAD33-*phoR*-HA, pUHE21-*mgtC*	This study
LJ73	14028s/pBAD33-*phoR*-HA, pUHE21	This study
KK149	*phoR*::*tetRA*^{R}	This study
EN949	*phoR* $^{Leu\ 421\ Ala}$	This study
EN836	*phoB*-HA::Cm^{R}	This study
EN842	*phoB*-HA /pUHE21	This study
EN843	*phoB*-HA /pUHE21-*mgtC*	This study
EN839	*phoB*-HA	This study
EN964	*phoR* $^{Leu\ 421\ Ala}$, *phoB*-HA::Cm^{R}	This study
EN966	*phoR* $^{Leu\ 421\ Ala}$, *phoB*-HA	This study
EN972	*phoR* $^{Leu\ 421\ Ala}$, *phoB*-HA /pUHE21	This study
EN973	*phoR* $^{Leu\ 421\ Ala}$, *phoB*-HA /pUHE21-*mgtC*	This study
EN991	*phoR* $^{Leu\ 421\ Gly}$	This study
EN1000	*phoR* $^{Leu\ 421\ Gly}$, *phoB*-HA::Cm^{R}	This study
EN1003	*phoR* $^{Leu\ 421\ Gly}$, *phoB*-HA	This study
EG19732	14028s/pUHE21	[1]
EG19733	14028s/pUHE21-*mgtC*	[1]
EN957	*phoR* $^{Leu\ 421\ Ala}$/pUHE21	This study
EN958	*phoR* $^{Leu\ 421\ Ala}$/pUHE21-*mgtC*	This study
SM063	14028s/pTGFP-*phoR*, pBAD33	This study
SM064	14028s/pTGFP-*phoR*, pBAD33-*mgtC*	This study
JH2	14028s/pTGFP- *phoR* $^{Leu\ 421\ Ala}$, pBAD33	This study
SM065	14028s/pTGFP- *phoR* $^{Leu\ 421\ Ala}$, pBAD33-*mgtC*	This study
SM076	14028s/pTGFP- *phoR* $^{Leu\ 421\ Gly}$, pBAD33-*mgtC*	This study

SM230	14028s/pTGFP, pBAD33-*mgtC*-FLAG	This study
SM257	14028s/pTGFP- *phoR*, pBAD33-*mgtC*-FLAG	This study
SM224	14028s/pTGFP- *phoR* $^{Leu\ 421\ Ala}$, pBAD33-*mgtC*-FLAG	This study
SM225	14028s/pTGFP- *phoR* $^{Leu\ 421\ Gly}$, pBAD33-*mgtC*-FLAG	This study
EL4	*mgtC*	[1]
EL551	*mgtC* $^{Ans\ 92\ Thr}$	[1]
SM085	*phoR* $^{Leu\ 421\ Ala}$, *mgtC*	This study
SM086	*phoR* $^{Leu\ 421\ Gly}$, *mgtC*	This study
EN296	*phoB*::Km^R	This study
KK10	*phoB*	This study
Escherichia coli		
DH5α	*fhuA2 lac(del)U169 phoA glnV44 Φ80' lacZ(del)M15 gyrA96 recA1 relA1 endA1 thi-1 hsdR17*	[5]
LJ25	DH5α/pKT25-*mgtR*	This study
LJ33	DH5α/pKT25-*phoR*	This study
LJ32	DH5α/pKT25-*phoU*	This study
LJ34	DH5α/pKT25-*pstB*	This study
SM001	DH5α/pKT25-*pstA*	This study
SM005	DH5α/pKT25-*phoR_TM_domain* (1-60)	This study
SM006	DH5α/pKT25-*phoR_PAS_domain* (61-180)	This study
SM007	DH5α/pKT25-*phoR_CA_domain* (181-431)	This study
SM009	DH5α/pKT25-*phoR_TM+PAS_domains* (1-180)	This study
SM066	DH5α/pKT25-*phoR 1-417*	This study
SM067	DH5α/pKT25-*phoR 1-418*	This study
SM068	DH5α/pKT25-*phoR 1-419*	This study
SM069	DH5α/pKT25-*phoR 1-420*	This study
LJ148	DH5α/pKT25-*phoR 1-421*	This study
LJ123	DH5α/pKT25-*phoR 1-422*	This study
LJ124	DH5α/pKT25-*phoR 1-423*	This study

LJ125	DH5α/pKT25-*phoR 1-424*	This study
LJ126	DH5α/pKT25-*phoR 1-425*	This study
LJ127	DH5α/pKT25-*phoR 1-426*	This study
SM070	DH5α/pKT25-*phoR* $^{Leu\ 421\ Ala}$	This study
SM071	DH5α/pKT25-*phoR* $^{Leu\ 421\ Ile}$	This study
SM072	DH5α/pKT25-*phoR* $^{Leu\ 421\ Val}$	This study
LJ154	DH5α/pKT25-*phoR* $^{Leu\ 421\ Gly}$	This study
SM050	DH5α/pTGFP-*phoR*	This study
JH18	DH5α/pTGFP- *phoR* $^{Leu\ 421\ Ala}$	This study
SM165	DH5α/pTGFP- *phoR* $^{Leu\ 421\ Gly}$	This study
BTH101	*F⁻, cya-854, recA1, endA1, gyrA96 (Nal^r), thi1, hsdR17, spoT1, rfbD1, glnV44(AS)*	[6]
EL643	BTH101/pUT18-*mgtC*	This study
LJ14	BTH101/pUT18-*mgtC*, pKT25-*mgtR*	This study
LJ27	BTH101/pUT18-*mgtC*, pKT25	This study
LJ41	BTH101/pUT18-*mgtC,* pKT25-*phoR*	This study
LJ40	BTH101/pUT18-*mgtC,* pKT25-*phoU*	This study
LJ39	BTH101/pUT18-*mgtC,* pKT25-*pstB*	This study
LJ38	BTH101/pUT18-*mgtC,* pKT25-*pstA*	This study
LJ42	BTH101/pUT18-*mgtC,* pKT25-*phoR_TM_domain* (1-60)	This study
LJ43	BTH101/pUT18-*mgtC,* pKT25-*phoR_PAS_domain* (61-180)	This study
LJ44	BTH101/pUT18-*mgtC,* pKT25-*phoR_CA_domain* (181-431)	This study
LJ62	BTH101/pUT18-*mgtC,* pKT25-*phoR_TM+PAS_domains* (1-180)	This study
LJ121	BTH101/pUT18-*mgtC,* pKT25-*phoR 1-417*	This study
LJ122	BTH101/pUT18-*mgtC,* pKT25-*phoR 1-418*	This study
LJ123	BTH101/pUT18-*mgtC,* pKT25-*phoR 1-419*	This study
SM167	BTH101/pUT18-*mgtC,* pKT25-*phoR 1-420*	This study
LJ115	BTH101/pUT18-*mgtC,* pKT25-*phoR 1-421*	This study

LJ142	BTH101/pUT18-*mgtC*, pKT25-*phoR 1-422*	This study
LJ141	BTH101/pUT18-*mgtC*, pKT25-*phoR 1-423*	This study
LJ144	BTH101/pUT18-*mgtC*, pKT25-*phoR 1-424*	This study
LJ152	BTH101/pUT18-*mgtC*, pKT25-*phoR 1-425*	This study
LJ147	BTH101/pUT18-*mgtC*, pKT25-*phoR 1-426*	This study
LJ155	BTH101/pUT18-*mgtC*, pKT25-*phoR* ^{Leu} $_{421\ Ala}$	This study
LJ145	BTH101/pUT18-*mgtC*, pKT25-*phoR* ^{Leu} $_{421\ Ile}$	This study
LJ146	BTH101/pUT18-*mgtC*, pKT25-*phoR* ^{Leu} $_{421\ Val}$	This study
LJ154	BTH101/pUT18-*mgtC*, pKT25-*phoR* ^{Leu} $_{421\ Gly}$	This study
LJ50	BTH101/pUT18-*mgtC* $^{Asn\ 92\ Thr}$, pKT25-*phoR*	This study
SM260	BTH101/pUT18-*mgtC* (130-231), pKT25-*phoR*	This study
**plasmids**		
pUHE21-2lacI^q	rep$_{pMBI}$ Ap^R *lacI*^{q}	[7]
p*mgtC*	pUHE21-*mgtC*	[8]
pBAD33	pACYC184 *ori* Cm^r	[9]
pKD3	repR$_{6K\gamma}$ Ap^R FRT Cm^R FRT	[10]
pKD4	repR$_{6K\gamma}$ Ap^R FRT Km^R FRT	[10]
pKD46	rep$_{pSC101}$^{ts} Ap^R P$_{araBAD}$ γ β exo	[10]
pCP20	rep$_{pSC101}$^{ts} Ap^R Cm^R *cI857* λP$_R$*flp*	[10]
pUT18	P$_{lac}$ ColEI*ori*Amp^r	[11]
pKT25	P$_{lac}$ p15A*ori*Km^r	[11]
pFCcGi	*rpsM*::*mCherry* and P$_{BAD}$::*gfpmut3a* promoter fusions in pFPV25.1, Ap^R	[12]
pTGFP	ColE1 *ori* Ap^R 'gfp	[101013]

Supplementary Table 2. Oligonucleotides used in this study

**Primers**	**Sequences (5′ to 3′)**
KHU131	CGGAATTCGGAGGAACGTATGTTAATGT
KHU132	CCCAAGCTTAACTATTATTGACTA
KHU195	GCTCTAGAGTGCTGGAACGGCTGTCATG
KHU196	CCCAAGCTTTTAGAGGCTAGCATAATCAGGAACATCATACGGATAATCGCTATTTTTGGCAATT
KHU355	GGAGCGTGAAGCGCAGACGCTCAGCCAGCAGAAACATACCTTAAGACCCACTTTCACATTTAAG
KHU356	CCGCTGGCTTATGGAAAGTTATACTTACGAAAGGCAATTACTAAGCACTTGTCTCCTGTTTAC
KHU351	GGCGGCCCCGGCGCTGTTAT
KHU352	AAACCACCAAACGTTGAATG
KHU596	AGCTTTGTGGCGCCGGAACGT
KHU597	ACGTTCCGGCGCCACAAAGCT
KHU425	TGTGTACGTTGAAGACCGGACGGT
KHU426	AGCGCGGGGATGCAACAGAGCACC
KHU435	AACGGTACGCGGGACAGGGTATCGTTTTTCGACCCGCTTTTATCCGTATGATGTTCCTGATTATGCTAGCCTCTAATGTAGGCTGGAGCTGCTTCG
KHU436	TTCTCCGCCAGAAACCTGTGTTCTACTGGCGGAAAAGGCACATATGAATATCCTCCTTAG

KHU450	CCGGAATTCCTTTACACTTTAAGCTTTTTATGTTTATGTTGTGTGGAGAGGGAGTATGACGCATGCTGGAACGGCTGTCATGGAAAA
KHU451	CCGGATCCATCGCTATTTTTGGCAATTAAAC
DE-phoB-F	ATGGCGCGGCATTGATAACTAACGACTAACAGGGCAAATTTGTAGGCTGGAGCTGCTTCG
DE-phoB-R	TTCTCCGCCAGAAACCTGTGTTCTACTGGCGGAAAAGGCACATATGAATATCCTCCTTAG
5-phoB-F	GGCGCGGCATTGATAACTAACGACTAA
3-phoB-R	GCCAGAAACCTGTGTTCTACTGGCGGA
KHU598	GCATTGATAGTGCATAATAGTTTTTT
KHU599	AAAAAACTATTATGCACTATCAATGC
KHU847	GTCAGGATCCCATGGAGGAACGTATGTTAAT
KHU848	GTCATGGTACCCGTTGACTATCAATGCTCCAGT
KHU849	CTAGAGATGAATCGCTCACCCGATAAAATCATCGCGCTGATATTTTTACTGATTAGCCTGTTGGTGTTGTGTTTAGCCCTCTGGCAAATCGTTTTCGGTAC
KHU850	CGAAAACGATTTGCCAGAGGGCTAAACACAACACCAACAGGCTAATCAGTAAAAATATCAGCGCGATGATTTTATCGGGTGAGCGATTCATCT

KHU881	GCTCTAGATACTTATTCTTCCAGAAAAAATGGAGGAACGTAT GTTAAT
KHU882	CCCAAGCTTTTACTTGTCATCGTCGTCCTTGTAGTCTTGACTAT CAATGCTCCAGT
KHU153	CGGGATCCGCTGGAACGGCTGTCATGGAAA
KHU154	GGGGTACCCCATCGCTATTTTTGGCAATTA
KHU155	CGGGATCCGGACAGTCTGAACCTTAATAAA
KHU156	GGGGTACCCCCTCTTTCGGATCTTTCCCCG
KHU157	CGGGATCCGGCTACGCTTGATATGCAGAAC
KHU158	GGGGTACCCCACCGTGTTTTTTCTTCGCGA
KHU159	CGGGATCCGAGTATGGTTGAAACTGCCCCG
KHU160	GGGGTACCCCACCGTAACGACCGGTGATAT
KHU187	CGGGATCCGCTGGAGCTGGTGCTCTGTTGC
KHU188	GGGGTACCCCCAAATTCCAGAAATGCCAGA
KHU189	CGGGATCCGCTGATTAAACGTTTTCGCAGT
KHU190	GGGGTACCCCCCCGGTATTGAGCACCAAA
KHU191	CGGGATCCGAACGTTAGCCATGAGTTGCGT
KHU192	GGGGTACCCCTAAACGTTCCGGCAGCACAA
KHU343	CGGGATCCGCGACAGGCGCCGCCGTTTTT
KHU344	GGGGTACCCCTTATTGACTATCAATGCTCC
KHU363	CGGGATCCGCTGGAACGGCTGTCATGGAAA

KHU463	GGGGTACCCCTCGCGTTCCTTTGCCAGGCG
KHU489	GGGGTACCCCCACAAAGCTAAATCGCGTTC
KHU490	GGGGTACCCCCAGCACAAAGCTAAATCGCG
KHU491	GGGGTACCCCCGGCAGCACAAAGCTAAATC
KHU492	GGGGTACCCCTTCCGGCAGCACAAAGCTAA
KHU493	GGGGTACCCCACGTTCCGGCAGCACAAAGC
KHU511	GGGGTACCCCTAAACGTTCCGGCAGCACAA
KHU539	GGGGTACCCCAAAGCTAAATCGCGTTCCTTT
KHU540	GGGGTACCCCGCTAAATCGCGTTCCTTTGCC
KHU541	GGGGTACCCCAAATCGCGTTCCTTTGCCAGG
KHU542	GGGGTACCCCACCCACAAAGCTAAATCGCG
KHU543	GGGGTACCCCAGCCACAAAGCTAAATCGCG
KHU544	GGGGTACCCCTTCCACAAAGCTAAATCGCG
KHU545	GGGGTACCCCAATCACAAAGCTAAATCGCG
KHU76	ATAAACAAATAGGGGTTCCG
KHU77	GTTTTCCGTATGTTGCATCA
KHU332	GTCCAACTTCCGCGACTCGG
KHU360	TGCTGCAAGGCGATTAAGTT
KHU484	GTATCACGAGGCCCTTTCGTCTTCA
KHU485	CGGCAACCGAGCGTTCTGAACAAAT
KHU566	GGCGTCACACTTTGCTATGCCATAG

KHU567	TTCACTTCTGAGTTCGGCATGGGGT
KHU574	GCC CAC TGC GGA ACG GGC GC
KHU579	AGCTTTGTGGGGCCGGAACGT
KHU580	ACGTTCCGGCCCCACAAAGCT
KHU608	CCCAGGCTTTACACTTTATG
KHQ015	CGCTGGGAAGCTGAGTTTG
KHQ016	CCAGGCCTCAACATCGTACA
Q-phoB-F	AGCCCGTAGAAGCCGAAGAT
Q-phoB-R	GGTCATGGCTTCACGTTTGA
Q-pstS-F	TGCGTACCACTGTCGCAACT
Q-pstS-R	GTATCCGCCCATTTGGCATA
Q-phnS-F	GCAAAAGAGCGCACGAATC
Q-phnS-R	GATGCGGTGTCGGTGTTAAA
Q-ugpB-F	ATCTGCTCTCCCAGCCCTTT
Q-ugpB-R	CGCTCTCAATTTCGCGGTAT
6970	CCAGCAGCCGCGGTAAT
6971	TTTACGCCCAGTAATTCCGATT
7530	CAGCCCGCGCACATTC
7531	CAGCCCGCGCACATTC

Supplementary Table 3. List of genes differentially expressed in the presence of the *mgtC* gene

(**a**) upregulated genes (≥4-fold)

Locus Tag	Old Locus Tag	Product	log2 Fold Change	Adjusted p-value
STM14_RS02210	STM14_0376	phosphoporin PhoE	4.82	3.90E-117
STM14_RS02755	STM14_0505	phosphonate ABC transporter permease	3.06	1.38E-06
STM14_RS02760	STM14_0506	phosphonate ABC transporter ATP-binding protein	2.5	1.35E-10
STM14_RS06355	STM14_1341	flagella synthesis protein FlgN	2.71	3.03E-26
STM14_RS06360	STM14_1342	negative regulator of flagellin synthesis	2.95	2.78E-24
STM14_RS06365	STM14_1343	flagella basal body P-ring formation protein FlgA	3.46	3.58E-34
STM14_RS06370	STM14_1345	flagellar biosynthesis protein FlgB	4.55	2.14E-84
STM14_RS06375	STM14_1346	flagellar basal body rod protein FlgC	4.84	9.64E-83
STM14_RS06380	STM14_1347	flagellar basal body rod modification protein FlgD	5.03	8.08E-145
STM14_RS06385	STM14_1348	flagellar hook protein FlgE	4.58	2.46E-270
STM14_RS06390	STM14_1349	flagellar basal body rod protein FlgF	4.44	1.21E-171
STM14_RS06395	STM14_1350	flagellar basal body rod protein FlgG	4.52	5.95E-165
STM14_RS06400	STM14_1351	flagellar L-ring protein	4.16	5.05E-66
STM14_RS06405	STM14_1352	flagellar P-ring protein	3.95	3.99E-78
STM14_RS06410	STM14_1353	peptidoglycan hydrolase FlgJ	3.82	6.53E-62
STM14_RS06415	STM14_1354	flagellar hook-associated protein 1	3.9	4.44E-134
STM14_RS06420	STM14_1355	flagellar hook-associated protein	3.61	7.87E-104
STM14_RS08840	STM14_1928	hypothetical protein	3.05	1.73E-35
STM14_RS08845	STM14_1929	virulence factor SrfB	2.88	3.27E-63
STM14_RS08850	STM14_1930	virulence effector SrfC	2.07	6.03E-34
STM14_RS09880	STM14_2174	flagellar brake protein YcgR	2.49	1.53E-14
STM14_RS10470	STM14_2326	flagellar biosynthesis protein FlhE	3.45	3.04E-07
STM14_RS10475	STM14_2327	flagellar biosynthesis protein FlhA	3.34	9.01E-52
STM14_RS10480	STM14_2328	flagellar biosynthetic protein FlhB	2.57	6.53E-11
STM14_RS10485	STM14_2330	protein phosphatase CheZ	3.17	2.40E-58
STM14_RS10490	STM14_2331	two-component system response regulator	3.55	6.14E-38
STM14_RS10495	STM14_2332	chemotaxis response regulator protein-glutamate methylesterase	4.02	4.81E-29
STM14_RS10500	STM14_2333	chemotaxis protein methyltransferase	3.79	2.24E-23
STM14_RS10505	STM14_2334	methyl-accepting chemotaxis protein II	4.8	6.50E-91
STM14_RS10510	STM14_2335	chemotaxis protein CheW	3.55	2.81E-49
STM14_RS10515	STM14_2336	chemotaxis protein CheA	3.36	4.23E-124
STM14_RS10520	STM14_2337	motility protein B	2.7	1.58E-27
STM14_RS10525	STM14_2338	motility protein A	2.51	8.06E-17
STM14_RS10580	STM14_2352	hypothetical protein	3.04	2.07E-10
STM14_RS10685	STM14_2373	protein FliZ	4.25	6.94E-40
STM14_RS10690	STM14_2374	RNA polymerase sigma factor FliA	4.39	2.51E-105
STM14_RS10700	STM14_2377	lysine-N-methylase	3.32	4.93E-46
STM14_RS10705	STM14_2378	flagellin	5.96	0

STM14_RS10710	STM14_2380	flagellar hook-associated protein 2	4.68	3.61E-170
STM14_RS10715	STM14_2381	flagellar protein FliS	4.04	3.93E-39
STM14_RS10720	STM14_2382	flagellar protein FliT	3.82	8.22E-34
STM14_RS10750	STM14_2388	flagellar hook-basal body protein FliE	4.63	4.74E-29
STM14_RS10755	STM14_2390	flagellar M-ring protein	3.79	8.75E-112
STM14_RS10760	STM14_2391	flagellar motor switch protein FliG	4.29	7.64E-79
STM14_RS10765	STM14_2392	flagellar assembly protein FliH	4.19	7.79E-64
STM14_RS10770	STM14_2393	flagellum-specific ATP synthase	4.18	2.56E-61
STM14_RS10775	STM14_2394	flagellar protein FliJ	3.83	2.74E-28
STM14_RS10780	STM14_2395	flagellar hook-length control protein	4.32	4.96E-111
STM14_RS10785	STM14_2396	flagellar basal body-associated protein FliL	3.69	6.27E-39
STM14_RS10790	STM14_2397	flagellar motor switch protein FliM	3.57	6.23E-71
STM14_RS10795	STM14_2398	flagellar motor switch protein FliN	3.53	2.02E-35
STM14_RS10800	STM14_2399	flagellar biosynthesis protein FliO	3.39	6.42E-29
STM14_RS10805	STM14_2400	flagellar biosynthetic protein FliP	2.79	6.42E-20
STM14_RS10810	STM14_2401	flagellar biosynthetic protein FliQ	3.16	7.11E-07
STM14_RS12780	STM14_2852	chemotaxis protein CheV	2.75	1.96E-29
STM14_RS16830	STM14_3817	methyl-accepting chemotaxis protein	2.66	2.02E-41
STM14_RS17155	STM14_3893	methyl-accepting chemotaxis protein	3.13	2.62E-39
STM14_RS18830	STM14_4281	sn-glycerol 3-phosphate ABC transporter permease	2.18	1.10E-32
STM14_RS18835	STM14_4282	sn-glycerol 3-phosphate ABC transporter permease	2.35	3.33E-34
STM14_RS18840	STM14_4283	sn-glycerol-3-phosphate-binding periplasmic protein UgpB	3.13	1.43E-140
STM14_RS18940	STM14_4305	methyl-accepting chemotaxis citrate transducer	2.2	1.22E-31
STM14_RS19110	STM14_4346	cyclic-guanylate-specific phosphodiesterase	3.47	2.61E-19
STM14_RS19885	STM14_4538	protein MgtC	6	0
STM14_RS20000	STM14_4568	hexose phosphate transporter	4.92	5.77E-194
STM14_RS20345	STM14_4648	phosphate transport system regulator PhoU	3.02	2.07E-151
STM14_RS20350	STM14_4649	phosphate ABC transporter ATP-binding protein	3.15	8.30E-154
STM14_RS20355	STM14_4650	phosphate transporter permease subunit PtsA	3.69	1.06E-142
STM14_RS20360	STM14_4651	phosphate ABC transporter permease	4.07	1.06E-177
STM14_RS20365	STM14_4652	phosphate ABC transporter substrate-binding protein	4.72	0
STM14_RS21385	STM14_4887	sulfate-binding protein	2.6	2.72E-110
STM14_RS23750	STM14_5445	carbon starvation protein	2.55	9.36E-19
STM14_RS23755	STM14_5446	methyl-accepting chemotaxis protein	3.89	3.18E-142

(**b**) downregulated genes (≥4-fold)

Locus Tag	Old Locus Tag	Product	log2 Fold Change	Adjusted p-value
STM14_RS05070	STM14_1039	arginine/ornithine ABC transporter substrate-binding protein	-2.82	0.000412
STM14_RS07050	STM14_1497	Virulence protein PagD	-2.11	0.00014
STM14_RS07060	STM14_1499	hypothetical protein	-2.36	4.93E-10
STM14_RS07065	STM14_1501	Virulence membrane protein PagC	-2.18	3.34E-62
STM14_RS10215	STM14_2257	hypothetical protein	-2.13	3.20E-06

STM14_RS10220	STM14_2258	disulfide bond formation protein B	-2.18	3.63E-16
STM14_RS14920	STM14_3352	VirG localization protein VirK	-2.25	6.69E-76
STM14_RS15370	STM14_3463	transcriptional regulator	-2.05	0.000833
STM14_RS21670	STM14_4956	N-acetyl-gamma-glutamyl-phosphate reductase	-2.06	3.76E-05
STM14_RS23420	STM14_5364	ornithine carbamoyltransferase	-2.78	4.21E-07

Supplementary References

1. Lee EJ, Pontes MH, Groisman EA. A Bacterial Virulence Protein Promotes Pathogenicity by Inhibiting the Bacterium's Own F_1F_o ATP Synthase. *Cell* **154**, 146-156 (2013).

2. Alix E, Blanc-Potard AB. Peptide-assisted degradation of the *Salmonella* MgtC virulence factor. *Embo J* **27**, 546-557 (2008).

3. Fields PI, Swanson RV, Haidaris CG, Heffron F. Mutants of *Salmonella typhimurium* that cannot survive within the macrophage are avirulent. *Proc Natl Acad Sci U S A* **83**, 5189-5193 (1986).

4. Fields PI, Groisman EA, Heffron F. A *Salmonella* locus that controls resistance to microbicidal proteins from phagocytic cells. *Science* **243**, 1059-1062 (1989).

5. Taylor RG, Walker DC, McInnes RR. *E. coli* host strains significantly affect the quality of small scale plasmid DNA preparations used for sequencing. *Nucleic Acids Res* **21**, 1677-1678 (1993).

6. Karimova G, Pidoux J, Ullmann A, Ladant D. A bacterial two-hybrid system based on a reconstituted signal transduction pathway. *Proc Natl Acad Sci U S A* **95**, 5752-5756 (1998).

7. Soncini FC, Vescovi EG, Groisman EA. Transcriptional autoregulation of the *Salmonella typhimurium phoPQ* operon. *J Bacteriol* **177**, 4364-4371 (1995).

8. Chamnongpol S, Groisman EA. Mg^{2+} homeostasis and avoidance of metal toxicity. *Mol Microbiol* **44**, 561-571 (2002).

9. Guzman LM, Belin D, Carson MJ, Beckwith J. Tight regulation, modulation, and high-level expression by vectors containing the arabinose P_{BAD} promoter. *J Bacteriol* **177**, 4121-4130 (1995).

10. Datsenko KA, Wanner BL. One-step inactivation of chromosomal genes in *Escherichia coli* K-12 using PCR products. *Proc Natl Acad Sci U S A* **97**, 6640-6645 (2000).

11. Karimova G, Ullmann A, Ladant D. Protein-protein interaction between *Bacillus stearothermophilus* tyrosyl-tRNA synthetase subdomains revealed by a bacterial two-hybrid system. *Journal of molecular microbiology and biotechnology* **3**, 73-82 (2001).

12. Figueira R, Watson KG, Holden DW, Helaine S. Identification of *salmonella* pathogenicity island-2 type III secretion system effectors involved in intramacrophage replication of *S. enterica* serovar typhimurium: implications for rational vaccine design. *mBio* **4**, e00065 (2013).
